# Comparative Efficacy and Safety of Thrombectomy Versus Thrombolysis for Large Vessel Occlusion in Acute Ischemic Stroke: A Systemic Review

**DOI:** 10.7759/cureus.72323

**Published:** 2024-10-24

**Authors:** Donald O Faletti, Opeyemi O Fakayode, Victor O Adedara, Azeez O Kuteyi, Charles A Adedara, Temiloluwa E Ogunmoyin, Jeffrey C Chen, Omolara Olasimbo, Susan A Aina, Grant U Alozie, Oluwatosin D Sadiku, Nate Nettagul, Anesia N Farrell, Boluwatife O Giwa

**Affiliations:** 1 Neurology, St. George's University School of Medicine, St. George's, GRD; 2 Medicine and Surgery, St. George's University School of Medicine, St. George's, GRD; 3 Medicine, St. George's University School of Medicine, St. George's, GRD; 4 Family Medicine, University of Mississippi Medical Center, Jackson, USA; 5 Medicine, Family Medicine, and Obstetrics, St. George's University School of Medicine, St. George's, GRD; 6 Neurology, University of Connecticut, Hartford, USA; 7 Internal Medicine, Temple University Hospital, Philadelphia, USA; 8 Cardiothoracic Surgery, St. George's University School of Medicine, St. George's, GRD; 9 Otolaryngology, St. George's University School of Medicine, St. George's, GRD; 10 Pediatrics and Internal Medicine, National Health Services West Midlands, Birmingham, GBR; 11 Medicine and Neurology, Russells Hall Hospital, Dudley, GBR

**Keywords:** alteplase, bridging therapy, large vessel occlusion, mechanical thrombectomy, modified rankin scale, stroke, thrombolysis

## Abstract

Acute ischemic stroke (AIS) is a common cause behind a significant number of people who develop disabilities or die worldwide. Most of the strokes that occur globally are attributed to AIS as a result of large vessel occlusions that typically occur in arteries like the internal carotid and middle cerebral arteries. Primary treatments for AIS are mechanical thrombectomy (MT) and intravenous thrombolysis (IVT), and the clinical scenario can dictate what method would provide the most optimal outcome for the patient. MT has a more favorable efficacy and safety profile but can be more technically challenging and time-consuming. This article conducts a comparison with regard to safety and efficacy between MT and IVT, which are the primary treatment methods for AIS.

The PubMed, Cochrane Library, Europe PubMed Central, Science Direct, and Google Scholar databases were used to search for relevant articles. This search was conducted from June 2024 to July 2024. The process involved examining the titles and abstracts of all relevant publications after which, the selected articles were read entirely to confirm eligibility. The Risk of Bias in Nonrandomized Studies of Interventions I tool was used to assess for bias in the articles selected.

The management of AIS involving IVT with or without MT is highly dependent on the clinical scenario. Nevertheless, MT alone has demonstrated better or comparable functional outcomes in patients compared to both bridging therapy (BT) and IVT alone. However, it is important to note that in select patient groups, such as those with large artery atherosclerosis, BT has been able to show better efficacy than MT alone. Given the significant burden of AIS on patient quality of life and healthcare spending, it is prudent to continue to explore newer thrombolytics and thrombectomy techniques.

## Introduction and background

Acute ischemic stroke (AIS) is a significant cause of disability and mortality globally. It is often a result of large vessel occlusions (LVOs), particularly in arteries like the internal carotid artery (ICA) and middle cerebral arteries [1]. Globally, AIS constituted 65% of the 89.13 million recorded strokes. In the United States alone, over 690,000 AISs are recorded annually, resulting in 140,000 deaths [2]. This leads to substantial disability and healthcare costs exceeding $70 billion [3,4] and, as such, remains a primary global health concern [5].

AIS is cerebral blood flow obstruction that leads to cerebral tissue damage. Focal and global neurological deficits such as dementia and functional impairments are very commonly observed after an AIS [1,6]. Rapid and effective treatment is crucial to prevent this to restore perfusion and minimize brain damage [7].

Primary treatments for AIS due to LVOs include mechanical thrombectomy (MT), which involves endovascular clot removal; intravenous thrombolysis (IVT), which uses pharmacological agents like tissue plasminogen activator to dissolve clots [2]; and bridging therapy (BT), a combination regimen of both treatment methods. This paper aims to compare the safety and efficacy of all treatment modalities based on recent scientific evidence and to provide guidance for medical professionals.

## Review

Methods

This review is a systematic review that evaluates the outcomes of MT, IVT, and BT for LVO strokes using the modified Rankin Scale (mRS) and safety outcomes. The methodology aligns with the principles of systematic reviews due to its comprehensive literature search, well-defined inclusion and exclusion criteria, and use of the Risk of Bias in Nonrandomized Studies of Interventions I (ROBINS-I) bias assessment tool.

The review begins with a comprehensive search strategy, where relevant articles were identified through five major databases: PubMed, Cochrane Library, Europe PubMed Central (PMC), Science Direct, and Google Scholar. The search used specific keywords such as "stroke", "large vessel occlusion", "modified Rankin Scale", "mechanical thrombectomy", "alteplase", "thrombolysis", and "bridging therapy". This broad search ensured the capture of relevant literature across various sources.

Studies included in the review had to meet several inclusion criteria: they must have been written in English, published between 2014 and 2024, peer-reviewed, conducted on human participants, and used the mRS and safety outcomes to compare MT or IVT with BT. Eligible studies included randomized clinical trials, prospective cohort studies, and retrospective cohort studies. The exclusion criteria ruled out studies with insufficient data, duplicates, gray literature, case reports, cross-sectional studies, and nonrandomized trials. These criteria helped ensure that only high-quality relevant studies were analyzed.

The ROBINS-I tool was employed to assess the quality of the studies [8]. This tool allowed for a detailed evaluation of bias across several domains. Of the identified studies, 13 were included in the review, with a general finding of low-to-moderate risk of bias. This assessment provides confidence in the reliability of the findings and ensures that the included studies are robust.

**Figure 1:**
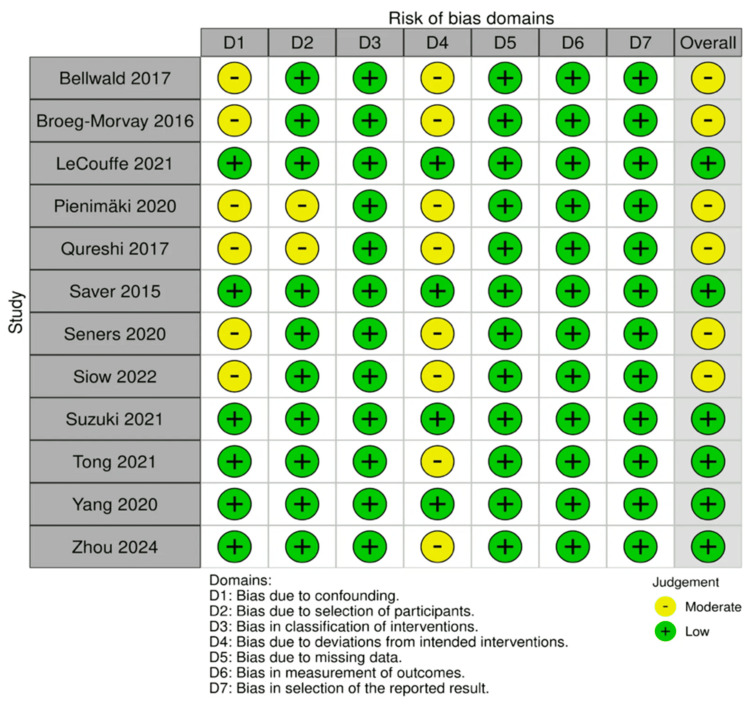
Risk-of-bias assessment for included studies using robvis. The overall risk of bias is presented as low (green) and moderate (yellow) robvis: Risk-Of-Bias VISualization Image credits: The image has been created by the authors

**Figure 2:**
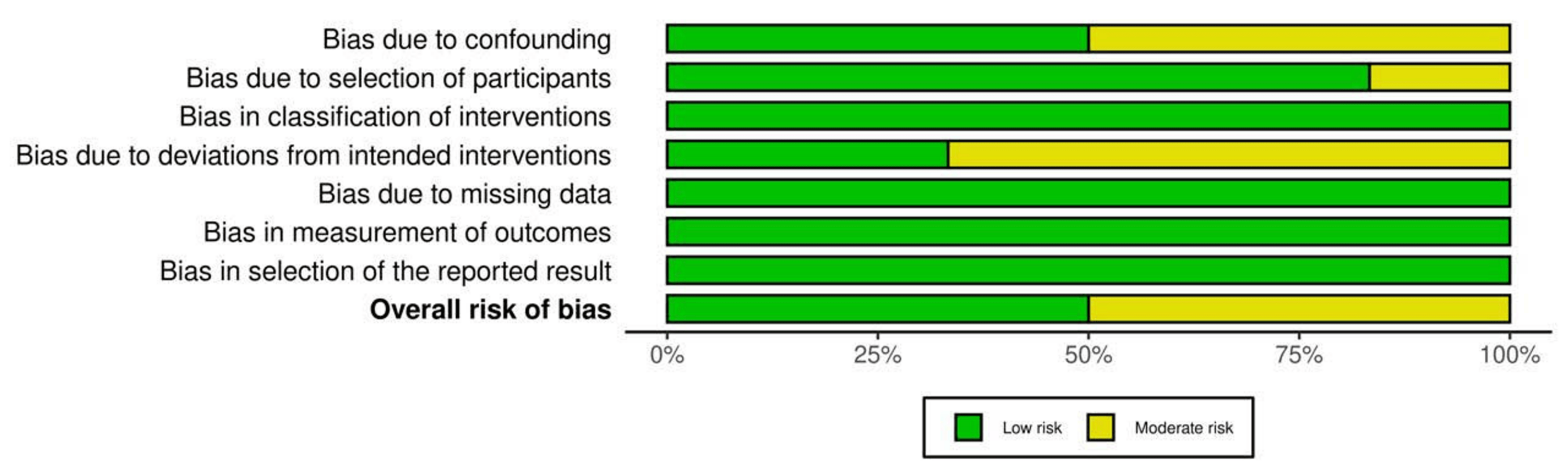
Risk-of-bias summary Image credits: The image has been created by the authors

Additionally, a risk-of-bias traffic light chart was used to visually represent the degree of bias in each study. This chart helps communicate the potential risks across various bias domains, with green, yellow, and red indicators representing low, moderate, and high risks, respectively. This visual tool aids in interpreting the overall quality of the evidence included in the review in Figures 1, 2.

This systematic review rigorously follows a transparent and predefined process, including a thorough literature search, strict eligibility criteria, and standardized bias assessment. It conforms to the Preferred Reporting Items for Systematic Reviews and Meta-Analyses guidelines and offers a detailed evaluation of MT, IVT, and BT for stroke management based on functional and safety outcomes.

Results

**Figure 3:**
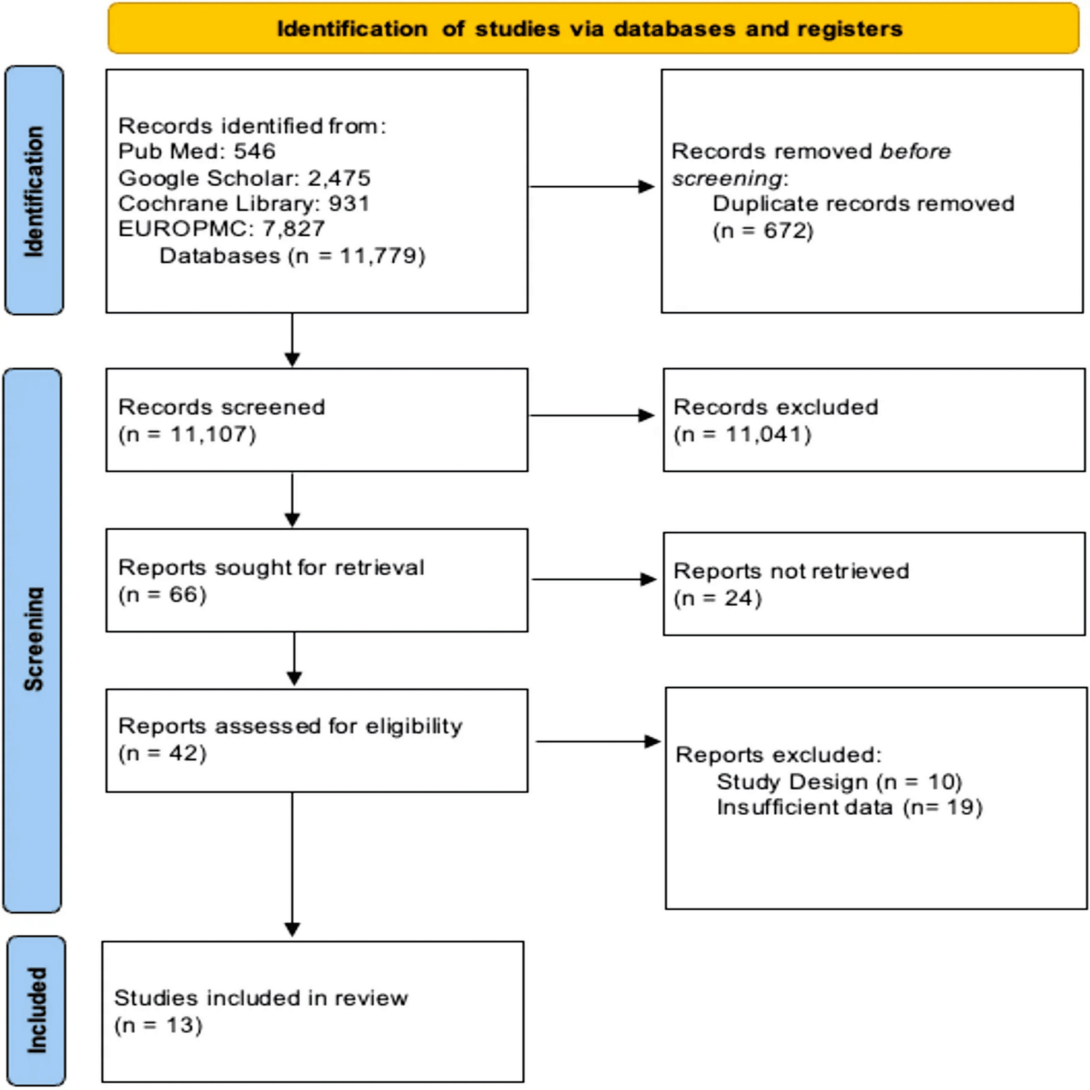
Flow diagram of the selection process based on the study's inclusion and exclusion criteria EuroPMC: Europe PubMed Central Image credits: The image has been created by the authors

A total of 11,779 publications were identified. Five hundred forty-six articles were from PubMed, 931 were from Cochrane Library, 7,827 were from Europe PMC, and 2,475 were from Google Scholar. Six hundred seventy-two duplicates were removed before screening. A total of 11,041 publications were excluded, and 24 publications could not be retrieved. After excluding 29 additional publications due to study design and insufficient data, 13 publications were ultimately used, as shown in Figure 3.

The chosen studies were published between 2015 and 2024 with 5,448 participants and representation from multiple countries, including France, Germany, the Netherlands, Finland, Singapore, the United Kingdom, the United States, Belgium, Switzerland, Sweden, Japan, and China. The studies primarily evaluated the efficacy and safety of MT alone compared to BT, as well as the efficacy and safety of IVT alone compared to BT for patients with AIS due to LVOs. Interventions included MT, such as stent retriever and aspiration thrombectomy, as well as alteplase.

An indirect comparison of MT and IVT was conducted using the efficacy and safety data. The primary outcome was functional outcome as measured by the mRS score at 90 days. The secondary outcomes evaluated included thrombolysis in cerebral infarction scores and National Institutes of Health Stroke Scale (NIHSS) score improvements. The main safety outcomes included mortality rates at 90 days and incidence of intracerebral hemorrhage (ICH) and symptomatic intracerebral hemorrhage (sICH). The results of these studies are presented in Table 1.

**Table 1:** Summary of studies comparing MT alone and BT in stroke patients A1/A2: anterior cerebral artery; ADL: activities of daily living; aICH: asymptomatic intracranial hemorrhage; BA: basilar artery; BT: bridging therapy; EQ-5D-5L: EuroQol 5-Dimension 5-Level; eTICI: extended thrombolysis in cerebral infarction; ICA: intracranial internal carotid; ICH: intracerebral hemorrhage; IMS III: Interventional Management of Stroke III; IVT: intravenous thrombolysis; LAA: large artery atherosclerosis; M1/M2: middle cerebral artery; mRS: modified Rankin Scale; MT: mechanical thrombectomy; mTICI: modified Thrombolysis in Cerebral Infarction; NIHSS: National Institutes of Health Stroke Scale; P1: posterior cerebral artery; PC: prospective cohort; RC: retrospective cohort; RCT: randomized clinical trial; SAH: subarachnoid hemorrhage; sICH: symptomatic intracerebral hemorrhage; TICI: thrombolysis in cerebral infarction

Study	Type of study (number of participants)	Country; centers	Interventions compared	Interventions used (mg/kg)	Primary outcome measured	Secondary outcomes measured	Occlusion sites	Results
LeCouffe et al. [9]	RCT (n = 539)	The Netherlands, Belgium, and France; multicenter	MT alone and BT	MT alteplase 0.9	mRS score at 90 days	eTICI score, NIHSS score improvement, mortality at 90 days, sICH incidence, ICH incidence, EQ-5D-5L score, and Barthel index score	ICA, M1, and M2	Functional outcome at 90 days was not significantly different between the MT-alone and BT groups. There was a slight but meaningful improvement in the NIHSS score at five to seven days or at discharge for the MT-alone group compared to the BT group, but no significant difference was seen at the 24-hour time point. Reperfusion, quality of life, ADL, ICH incidence, sICH incidence, and mortality at 90 days were not significantly different between the two groups
Zi et al. [10]	RCT (n = 234)	China; multicenter	MT alone and BT	MT alteplase 0.9	mRS score at 90 days	eTICI score, NIHSS score improvement, mortality at 90 days, sICH incidence, ICH incidence, and EQ-5D-5L score	Proximal anterior circulation	Functional outcome at 90 days was not significantly different between the MT-alone and BT groups. The MT-alone group was associated with a lower ICH incidence. Reperfusion, quality of life, NIHSS score improvement, sICH incidence, and in-hospital mortality were not significantly different between the two groups
Siow et al. [11]	RCT (n = 322)	Germany, United Kingdom, Taiwan, Sweden, and Singapore; multicenter	MT alone and BT	MT alteplase 0.9	mRS score at 90 days	mTICI score, in-hospital mortality, and sICH incidence	BA	Functional outcome at 90 days was not significantly different between the MT-alone and BT groups. Reperfusion, sICH incidence, and in-hospital mortality were not significantly different between the two groups. In a subgroup analysis for patients with LAA, BT was associated with superior functional outcomes at 90 days and in-hospital mortality compared to MT alone. There were no significant differences in reperfusion and sICH incidence between the groups in subgroup analysis
Suzuki et al. [12]	RCT (n = 204)	Japan; multicenter	MT alone and BT	MT alteplase 0.6	mRS score at 90 days	mTICI score, mortality at 90 days, sICH incidence, and ICH incidence	ICA, M1, and M2	Functional outcome at 90 days was not significantly different between the MT-alone and BT groups. The MT-alone group was associated with a lower ICH incidence. Reperfusion, sICH incidence, and mortality at 90 days were not significantly different between the two groups
Saver et al. [13]	RCT (n = 196)	United States and Europe; multicenter	IVT alone and BT	Stent retriever thrombectomy alteplase (unspecified dose)	mRS score at 90 days	mTICI score, NIHSS improvement, mortality at 90 days, and sICH incidence	ICA, M1, and M2	BT was associated with a higher functional outcome at 90 days, reperfusion, and NIHSS score improvement than IVT alone. sICH incidence and mortality in 90 days were not significantly different between the two groups. The study was stopped early because of efficacy
Yang et al. [14]	RCT (n = 656)	China; multicenter	MT alone and BT	MT alteplase 0.9	mRS score at 90 days	eTICI score, NIHSS improvement, mortality at 90 days, aICH incidence, sICH incidence, EQ-5D-5L score, and Barthel index	ICA, M1, and M2	Functional outcome at 90 days was not significantly different between the MT-alone and BT groups. The BT group was associated with better reperfusion before thrombectomy. Final reperfusion, quality of life, ADL, NIHSS score improvement, aICH incidence, sICH incidence, and mortality in 90 days were not significantly different between the two groups
Seners et al. [15]	RC (n = 598)	France; multicenter	IVT alone and BT	MT IVT (not specified)	mRS score at 90 days	eTICI score, sICH incidence, and ICH incidence	ICA, M1, and M2	Functional outcome at 90 days was not significantly different between the IVT-alone and BT groups. The BT group was associated with higher ICH and sICH incidences. Reperfusion was not significantly different between the two groups. For both “as treated” and “per-protocol” analyses, BT is associated with a better functional outcome in M1 strokes, while IVT alone is associated with a better functional outcome in M2 strokes. However, there is no significant difference between the therapies when both segments are combined
Pienimäki et al. [16]	RC (n = 106)	Finland; single center	MT and BT	Stent retriever thrombectomy alteplase 0.9	mRS score at 90 days	mTICI score, mortality at 90 days, and ICH incidence	ICA, M1, M2, and M3	Functional outcome at 90 days was not significantly different between the MT-alone and BT groups, although MT alone achieved a better rate of excellent functional outcomes (mRS 0-1) at 90 days. Reperfusion, ICH incidence, and mortality in 90 days were not significantly different between the two groups
Bellwald et al. [17]	RC (n = 360)	Germany and Switzerland; dual center	MT alone and BT	MT alteplase 0.6 and 0.9	mRS score at 90 days	TICI score, mortality, sICH incidence, and aICH incidence	ICA, M1, and M2	Functional outcome at 90 days was not significantly different between the MT-alone and BT groups. BT was associated with a higher mortality rate in patients with ICA strokes. Reperfusion, aICH incidence, and sICH were not significantly different between the two groups
Broeg-Morvay et al. [18]	RC (n = 422)	Switzerland; multicenter	MT alone and BT	MT alteplase 0.6 and 0.9	mRS score at 90 days	TICI score, NIHSS improvement, mortality at 90 days, sICH incidence, and aICH incidence	ICA, M1, and M2	Functional outcome at 90 days was not significantly different between the MT-alone and BT groups. In the matched-pairs analysis, MT alone was associated with a lower aICH incidence and lower mortality. There was a slightly better improvement in NIHSS scores in MT compared to BT (p = 0.049). Reperfusion and sICH were not significantly different between the two groups
Tong et al. [19]	PC (n = 1,026)	China; multicenter	MT alone and BT	MT alteplase 0.9	mRS score at 90 days	mTICI score, NIHSS improvement, 90-day mortality rate, sICH incidence, and ICH incidence	ICA, M1, M2, A1, A2, P1, and BA	Functional outcome at 90 days was not significantly different between the MT-alone and BT groups. MT alone was associated with a lower sICH incidence. Reperfusion, NIHSS scores improvement, and ICH were not significantly different between the two groups
Qureshi et al. [20]	RC (n = 51)	United States; multicenter	IVT alone and BT	MT alteplase 0.9	mRS score at 90 days	Mortality at 90 and 120 days, EQ-5D, and Barthel index score	M2	Functional outcome at 90 days was not significantly different between the IVT-alone and BT groups. BT was associated with lower mortality. Quality of life and ADL were not significantly different between the two groups. The IMS III trial had very limited use of stent retrievers as these devices were not approved for use in the United States during the early part of the trial
Zhou et al. [21]	RC (n = 734)	China; multicenter	IVT alone and BT	MT alteplase (unspecified dose)	mRS score at 90 days	Recanalization at 24 hours, sICH incidence, and mortality at 90 days	M2	Functional outcome at 90 days was not significantly different between the IVT-alone and BT groups. BT was associated with a greater percentage of recanalization at 24 hours. sICH incidence and mortality were not significantly different between the two groups. However, SAH incidence was higher for BT

Regarding efficacy, MT alone was equivalent to BT in achieving a favorable functional outcome in patients with AIS due to LVOs across most of the reviewed studies [9]. However, BT resulted in a superior functional outcome within certain subgroups, such as those with large artery atherosclerosis (LAA) [11]. Additionally, Yang et al. reported a higher rate of successful reperfusion before thrombectomy in patients receiving BT [14].

The safety profile for MT alone was generally more favorable compared to BT, showing a similar or lower incidence of ICH and sICH, as well as a lower incidence of intraprocedural embolization [10,16]. However, in Broeg-Morvay et al.'s matched-pair analysis, MT alone was associated with a higher risk of aICH [18]. Moreover, MT alone and BT were generally associated with similar mortality rates and improvement in NIHSS scores [9,10,17].

Regarding the efficacy of IVT alone compared to BT, IVT alone generally achieved lower functional outcomes, reduction in disability, functional independence, lower degrees of NIHSS improvement, and lower recanalization and reperfusion rates compared with BT [13]. However, Seners et al. reported a superior functional outcome for IVT in patients with AIS due to occlusions in the M2 segment. At the same time, BT was associated with a better functional outcome in M1 strokes in the "as treated" and "per-protocol" analyses [15]. The safety profile for IVT alone compared to BT generally demonstrated similar mortality; however, Seners et al. reported a higher incidence of ICH and sICH in patients treated with BT than those treated with IVT alone [13,15].

Discussion

Stroke is one of the leading causes of serious long-term disability in the United States and is the fifth leading cause of death when considered separately from other cardiovascular diseases [22]. Evaluating the efficacy and safety of interventions used to manage AIS is crucial to reducing the burden on the healthcare system. Because of the high disability and mortality rate associated with AIS due to LVOs, quick and informative choices must be made regarding the reperfusion of the blocked vessel. MT and IVT are the mainline treatments for AIS, providing essential vessel reperfusion. However, while IVT is part of the standard treatment for AIS, not all patients are eligible for its use due to contraindications such as presenting with a mild nondisabling stroke (NIHSS score <5), acute bleeding diathesis, and history of ICH, among others [23]. Additionally, not all patients may require unnecessary exposure to MT, which is reserved for those over 18 years of age presenting with an acute large artery occlusion of the anterior cerebral circulation or ICA [23,24].

To minimize risks, reduce treatment costs, and ensure effective treatment, evaluation of these interventions used alone and in combination is essential. Since 2015, MT has been implemented into the standard treatment of AIS, particularly for patients with LVOs. Since then, research has consistently demonstrated that the addition of MT in AIS treatment is highly beneficial and cost-effective, as shown in various studies across Europe, where MT has produced over 100,000 additional quality-adjusted life years (95% uncertainty interval, 65,180-149,085) and saved approximately $981 million and $1.7 billion in costs in health and social costs from 2017 and 2021, respectively [25].

MT used alone or in combination with IVT is associated with a favorable functional outcome comparable to or superior to IVT alone [13]. This suggests that when patients are not eligible for IVT or IVT is not readily available, MT alone can still be effective. However, the efficacy of MT appears to vary with the anatomical location of the occlusion. For instance, patients treated with MT for LVOs in the anterior circulation achieved more favorable outcomes in the studies reviewed. Conversely, IVT alone demonstrated superior efficacy in smaller posterior circulation strokes [15].

Combining MT and IVT was shown to be advantageous in specific subgroups, such as those with LAA [14]. Additional research, outside of those included within this systematic review, such as those by Ballout et al. and Sun et al., further supports these findings. They attribute the slightly favorable outcomes to better pretreatment collaterals in patients with underlying LAA than those with cardioembolic AIS causes [26,27]. This finding reveals the nuanced role of patient selection and occlusion characteristics in treatment outcomes.

Safety profiles generally favored MT alone compared to BT and IVT alone, particularly regarding a lower incidence of ICH. This safety advantage can be a significant factor in treatment choice, given the potential hemorrhagic complications associated with thrombolytic therapy [28].

Several limitations in the current literature should be noted. The studies included in this review span a decade, and during this time, substantial developments in the function and protocol of MT and thrombolysis, as well as improved imaging techniques. Notably, the IMS III trial, analyzed by Qureshi et al., had limited use of stent retrievers as these devices were not approved for use in the United States during the early part of the trial [20]. Additionally, the variation in study design, patient populations, and study protocols limits the generalizability of findings. The studies conducted by Yang et al., Tong et al., and Zhou et al. primarily included a population of Asian patients [14,19,21]. The higher prevalence of intracranial atherosclerotic disease in Asian populations compared to Western populations may potentially affect the generalizability of the findings to other ethnic groups [12,29].

## Conclusions

Treatment options for AIS due to LVOs vary, but the efficacy and safety of these options are of significant concern. AIS results in severe functional deficits affecting thousands of individuals. It is, therefore, imperative to establish a standard of care for patients to prevent these severe complications. Our findings in this review indicate that, in general, MT, used alone or in combination with IVT, is associated with better functional outcomes and a favorable efficacy and safety profile, especially in anterior circulation occlusions. Additionally, while MT alone demonstrated comparable and occasionally better effectiveness than BT, BT showed greater efficacy than MT alone in some patient groups, such as those with underlying LAA.

It is also essential to highlight the nuanced decision-making process that goes into treating AIS patients and the limitations of literature on this topic, factors like study design, patient selection, population size, and ever-evolving medical protocols. Future studies should focus on refining patient selection criteria, and considering innate and pathological differences in different populations is imperative. Furthermore, the exploration of newer thrombolytic agents and thrombectomy techniques will be essential for reducing the global burden of stroke-related disability and mortality.
